# Supplementary material for: Effects of Combined Upper and Lower Limb Plyometric Training Interventions on Physical Fitness in Athletes: A Systematic Review with Meta-Analysis
Source: Int J Environ Res Public Health. 2022 Dec 28;20(1):482. doi: 10.3390/ijerph20010482 (PMC9819760; doi:10.3390/ijerph20010482)
Supplement: Supplementary file 1 [file ijerph-20-00482-s001.zip › Table S1. Search strategy.pdf]

Table S1. Detailed search strategy.

Search on 18 August 2022

Databases	search strategy	Results
Pubmed (1946 – August 2022)	("plyometric training" [Title/Abstract] OR "plyometric exercise*" [Title/Abstract] OR "stretch-shortening cycle" [Title/Abstract] OR "stretch-shortening exercise*" [Title/Abstract] AND ("player*" [Title/Abstract] OR "athlete*"[Title/Abstract] OR "sportsman*" [Title/Abstract]) OR "sportswoman*" [Title/Abstract]) OR "sportsperson*" [Title/Abstract])	531
Web of Science (1945 – August 2022)	(AB=("plyometric training" OR "plyometric exercise*" OR "stretch-shortening cycle" OR "stretch-shortening exercise*")) AND AB=("player*" OR "athlete*" OR "sportsman*" OR "sportswoman*" OR "sportsperson*")	615
(SPORTDicus) (1984 - August 2022)	AB ("plyometric training" OR "plyometric exercise*" OR "stretch-shortening cycle" OR "stretch-shortening exercise*") AND AB ("player*" OR "athlete*" OR "sportsman*" OR "sportswoman*" OR "sportsperson*")	661
Scopus (2004 – August 2022)	(TITLE-ABS-KEY("plyometric training" OR "plyometric exercise*" OR "stretch-shortening cycle" OR "stretch-shortening exercise*") AND TITLE-ABS-KEY("player*" OR "athlete*" OR "sportsman*" OR "sportswoman*" OR "sportsperson*"))	1124
Total		2870
