# Supplementary material for: Effects of Combined Upper and Lower Limb Plyometric Training Interventions on Physical Fitness in Athletes: A Systematic Review with Meta-Analysis
Source: Int J Environ Res Public Health. 2022 Dec 28;20(1):482. doi: 10.3390/ijerph20010482 (PMC9819760; doi:10.3390/ijerph20010482)
Supplement: Supplementary file 1 [file ijerph-20-00482-s001.zip › Table S2. Data for meta-analysis.pdf]

The data used for meta-analysis

Table S2. Performances (mean, standard deviation and number of players) in physical fitness tests.

References	Tests	Experimental (pre-test)			Control (pre-test)			Experimental (pos-test)			Control (pos-test)		
		Mean	SD	n	Mean	SD	n	Mean	SD	n	Mean	SD	n
**Muscle Power**													
**MBT (m)**													
Santos and Janeira. 2011 (2 ULLPT/week) [52]	MBT	3.43	0.4	14	3.1	0.4	10	3.94	0.4	14	3.27	0.4	10
Santos and Janeira. 2011 (1 ULLPT/week) [52]	MBT	3.77	0.31	7	3.27	0.35	7	4.17	0.29	7	3.19	0.47	7
Sadeghi et al. 2013 [54]	SMBT	1.83	0.41	12	1.9	0.43	12	2.02	0.28	12	1.95	0.26	12
Pereira et al. 2015 [57]	MBT	7.51	15.2	10	6.7	94.2	10	7.9	14.3	10	6	43.8	10
Ramírez-Campillo et al. 2016 (female) [58]	MBT	4.55	0.35	19	4.62	0.47	19	4.86	0.38	19	4.59	0.39	19
Ramírez-Campillo et al. 2016 (male) [58]	MBT	6.16	0.53	21	6.18	0.52	21	6.46	0.54	21	6.19	0.53	21
Idrizovic et al. 2018 [62]	MBT	6.1	0.5	13	5.8	0.7	17	7.7	0.7	13	6.4	0.9	17
Hammami et al. 2019 [32]	MBT	2.9	0.31	21	2.86	0.31	20	3.7	0.31	21	3.06	0.31	20
**CMJ(cm)**													
Santos and Janeira. 2011 (2 ULLPT/week) [52]	CMJ	30.33	4.3	14	30.76	5.1	10	34.52	5	14	28.4	4	10
Santos and Janeira. 2011 (1 ULLPT/week) [52]	CMJ	34.92	4.5	7	28.4	4	7	39.33	5.5	7	28.68	4.9	7
Chelly et al. 2014 [56]	CMJ	42	4	12	41	3	11	46	4	12	42	3	11
Pereira et al. 2015 [57]	CMJ	26.9	4.5	10	25	3.7	10	32.3	9	10	25.8	3.7	10
Hall et al. 2016 [59]	CMJ	43.5	6.1	10	45.1	5.8	10	45.3	5.8	10	45.3	5.5	10
Ramírez-Campillo et al. 2016 (female) [58]	CMJ	26.7	5.5	19	26.6	4.8	19	29.4	5.8	19	26.6	4.3	19
Ramírez-Campillo et al. 2016 (male) [58]	CMJ	35.3	3.3	21	33.2	3.9	21	37.6	4	21	32.8	3.8	21
Idrizovic et al. 2018 [62]	CMJ	42.2	6	13	41.7	4.3	17	49.5	7	13	45.1	5.1	17
Hammami et al. 2019 [32]	CMJ	20.8	4.7	21	21.6	4.2	20	26.7	4.7	21	23	4.2	20

The data used for meta-analysis

Table S2. (Continued).													
Hammami et al. 2020 [63]	CMJ	24.2	1.6	17	24	2	17	29.3	1.8	17	24.9	1.8	17
Canlı and Bayru. 2020 [31]	CMJ	36.4	3.62	15	36.1	6.62	15	39.6	3.58	15	37.2	6.65	15
**CMJa (cm)**													
Santos and Janeira. 2011 (2 ULLPT/week) [52]	CMJa	35.65	4.4	14	36.12	4.8	10	40.59	4.2	14	34.32	4.8	10
Santos and Janeira. 2011 (1 ULLPT/week) [52]	CMJa	41.59	2.8	7	34.32	4.8	7	44.24	4.3	7	34.97	5.6	7
Karadenizli et al. 2016 [60]	CMJa	37.5	5.6	14	36.2	5.3	12	41.6	7.3	14	37.3	8.9	12
Ramírez-Campillo et al. 2016 (female) [58]	CMJa	30.3	6.5	19	29.2	5.5	19	32.6	6.5	19	28.8	5.1	19
Ramírez-Campillo et al. 2016 (male) [58]	CMJa	41	3.8	21	37.5	4.4	21	44.3	3.9	21	37.6	40	21
Hammami et al. 2019 [32]	CMJa	25.7	1.5	21	25.5	1.5	20	32.1	1.5	21	27.9	1.5	20
Hammami et al. 2020 [63]	CMJa	25.3	1.6	17	25.3	2.1	17	30.4	2.1	17	25.8	2.1	17
**SJ (cm)**													
Santos and Janeira. 2011 (2 ULLPT/week) [52]	SJ	25.17	3.5	14	22.7	4.3	10	29.15	4.1	14	20.74	3.9	10
Santos and Janeira. 2011 (1 ULLPT/week) [52]	SJ	29.5	3.9	7	20.74	3.9	7	31.9	4.7	7	21.96	3.5	7
Chelly et al. 2014 [56]	SJ	39	4	12	39	3	11	44	4	12	40	3	11
Hammami et al. 2019 [32]	SJ	19.6	3.5	21	19.3	3.6	20	25.3	3.5	21	21	3.6	20
Hammami et al. 2020 [63]	SJ	22.4	1.7	17	22.8	2.1	17	26.4	1.9	17	23.8	1.6	17
**Muscle Strength**													
**Upper body**													
Sharma and Multani. 2012 [53]	Handgrip (pounds)	70.29	23.03	20	61.08	24.73	20	74.46	23.54	20	61.23	24.21	20
Behringer et al. 2013 [55]	Chest press (kg)	20.4	7.6	12	20.6	7.7	12	26.3	11.1	12	21.5	7.2	12
Uzun and Karakoc. 2017 [61]	Handgrip (kg)	43.89	5.25	15	46.33	8.23	15	47.5	3.61	15	46.98	9.58	15
Hammami et al. 2019 [32]	Handgrip (N)	174	23	21	175	23	20	246	23	21	186	23	20

The data used for meta-analysis

Table S2. (Continued).

Canli and Bayru. 2020 [31]	Bench press (kg)	40.9	6.34	15	40.5	7.69	15	43.4	6.9	15	39.9	7.63	15
Kurniawan et al. 2021 (active recovery) [64]	Handgrip (kg)	42.3	4.51	11	41.5	4.54	5	46.2	5.25	11	46.8	6.08	5
Kurniawan et al. 2021 (passive recovery) [64]	Handgrip (kg)	41.1	4.97	11	41.5	4.54	6	45.9	5.45	11	46.8	6.08	6
**Lower body**													
Sharma and Multani. 2012 [53]	Calf strength (s)	110.55	81.57	20	196.25	141.97	20	205.35	94.36	20	196.15	133.03	20
Behringer et al. 2013 [55]	Leg press (kg)	122.7	26	12	109.3	22.2	12	142.3	19.3	12	121.3	23.5	12
Uzun and Karakoc. 2017 [61]	Leg strength (kg)	117.46	26.75	15	127.78	46.3	15	125.56	24.18	15	127.03	41.46	15
Canli and Bayru. 2020 [31]	Leg press (kg)	156.2	26.41	15	120.7	35.24	15	177.26	27.63	15	136.4	34.49	15
**Linear Sprint Speed**													
**5 m**													
Chelly et al. 2014 [56]	5 m (m.$^{-1}$)	5.56	0.33	12	5.56	0.7	11	6.7	0.39	12	5.79	0.69	11
Hammami et al. 2019 [32]	5 m	1.3	0.11	21	1.26	0.06	20	1.21	0.11	21	1.22	0.06	20
Hammami et al. 2020 [63]	5 m	1.25	0.06	17	1.28	0.05	17	1.12	0.05	17	1.24	0.05	17
**20 m**													
Idrizovic et al. 2018 [62]	20 m	3.8	0.3	13	4	0.3	17	3.6	0.2	13	4	0.1	17
Hammami et al.2019 [32]	20 m	3.86	0.33	21	3.79	0.17	20	3.49	0.33	21	3.71	0.17	20
Hammami et al. 2020 [63]	20 m	3.77	0.05	17	3.75	0.05	17	3.56	0.05	17	3.68	0.07	17
**30 m**													
Karadenizli et al. 2016 [60]	30 m	5.38	0.23	14	5.44	0.25	12	4.93	0.2	14	5.41	0.47	12
Ramírez-Campillo et al. 2016 (female) [58]	30 m	5.69	0.31	19	5.72	0.28	19	5.4	0.32	19	5.82	0.31	19
Ramírez-Campillo et al. 2016 (male) [58]	30 m	5.05	0.17	21	5.05	0.18	21	4.79	0.18	21	5.05	0.12	21
Hammami et al. 2019 [32]	30 m	5.71	0.4	21	5.56	0.27	20	4.52	0.4	21	5.09	0.27	20
Hammami et al. 2020 [63]	30 m	4.64	0.05	17	5.54	0.05	17	4.28	0.07	17	5.49	0.07	17

The data used for meta-analysis

Table S2. (Continued).

Kurniawan et al. 2021 (active recovery) [64]	30 m	4.59	0.31	11	4.68	0.36	5	4.32	0.43	11	4.71	0.31	5
Kurniawan et al. 2021 (passive recovery) [64]	30 m	4.84	0.61	11	4.68	0.36	6	4.47	0.47	11	4.71	0.31	6
**Agility (s)**													
Sadeghi et al. 2013 [54]	Agility shuttle run	7.09	0.42	12	7.03	0.42	12	5.98	0.31	12	6.8	0.57	12
Karadenizli. 2016 [60]	Illinois-agility Test	16.02	0.23	14	16.88	0.86	12	16.02	0.38	14	16.76	0.49	12
Ramírez-Campillo et al. 2016 (female) [58]	Illinois-agility test	19.48	0.9	19	19.79	1	19	18.73	1	19	19.93	0.9	19
Ramírez-Campillo et al. 2016 (male) [58]	Illinois-agility test	17.72	0.7	21	17.55	0.6	21	17.32	0.7	21	17.65	0.7	21
Hammami et al. 2019 [32]	Modified Illinois test	13.88	0.46	21	13.88	0.49	20	12.78	0.47	21	13.5	0.49	20
Hammami et al. 2020 [63]	Modified Illinois test	13.07	0.07	17	13.11	0.39	17	12.10	0.09	17	13	0.39	17
**Flexibility (cm)**													
Sadeghi et al. 2013 [54]	Sit and reach	14.01	7.29	12	13.27	6.69	12	18.18	7.89	12	13.45	7.03	12
Karadenizli et al. 2016 [60]	Sit and reach	26.61	6.32	14	25.77	5.3	12	30.21	8.15	14	29.55	6.18	12
Uzun and Karakoc. 2017 [61]	Sit and reach	26.73	7.09	15	19.66	10.8	15	28.6	7.17	15	18.86	10.72	15
Idrizovic et al. 2018 [62]	Sit and reach	52	4.5	13	49.9	7	17	56.6	4.5	13	50.7	5.9	17
Kurniawan et al. 2021 (active recovery) [64]	Sit and reach	17.5	3.18	11	21.7	3.88	5	22.6	4.41	11	21.3	3.52	5
Kurniawan et al. 2021 (passive recovery) [64]	Sit and reach	21.3	4.03	11	21.7	3.88	6	23.4	4.46	11	21.3	3.52	6
**Balance**													
**Static**													
Karadenizli et al. 2016 [60]	Balance-unipedal test (mm^2)	1581.84	581.69	14	1558.62	487.83	12	1483.21	418.32	14	1510.19	396.21	12
Sharma and Multani. 2012 [53]	Single leg stance test (s)	185.25	164.31	20	246.8	150.93	20	344.90	148.64	20	240.65	141.46	20
Hammami et al. 2019 [32]	Stork balance test (s)	2.28	0.68	21	2.07	0.56	20	2.37	0.68	21	2.37	0.56	20

The data used for meta-analysis

Table S2. (Continued).

Hammami et al. 2020 [63]	Stork balance test (s)	2.33	1.16	17	2.61	1.16	17	3.37	1.05	17	3.92	1.93	17
**Dynamic**													
Karadenizli et al. 2016 [60]	Balance-bipedal Slalom Test (%)	0.4	0.1	14	0.4	0.2	12	0.1	0.1	14	0.1	0.1	12
Hammami et al. 2019 [32]	Y balance test (cm)	63.8	5.8	21	62.2	6.7	20	65.8	5.8	21	63.9	6.7	20
Hammami et al. 2020 [63]	Y balance test (cm)	75	7	17	73	9	17	79	8	17	79	9	17

MBT, medicine ball throw; SMBT, seat medicine ball throw; CMJ, countermovement jump; CMJa, countermovement jump with arm swing; n, number of players/athletes measured; SD, standard deviation; SJ, squat jump.
